# Toward on-the-fly trajectory optimization for C-arm CBCT under strong kinematic constraints

**DOI:** 10.1371/journal.pone.0245508

**Published:** 2021-02-09

**Authors:** Sepideh Hatamikia, Ander Biguri, Gernot Kronreif, Michael Figl, Tom Russ, Joachim Kettenbach, Martin Buschmann, Wolfgang Birkfellner

**Affiliations:** 1 Austrian Center for Medical Innovation and Technology, Wiener Neustadt, Austria; 2 Center for Medical Physics and Biomedical Engineering, Medical University of Vienna, Vienna, Austria; 3 Institute of Nuclear Medicine, University College London, London, United Kingdom; 4 Computer Assisted Clinical Medicine, Medical Faculty Mannheim, Heidelberg University, Heidelberg, Germany; 5 Institute of Diagnostic and Interventional Radiology and Nuclear Medicine, Landesklinikum, Wiener Neustadt, Austria; 6 Department of Radiation Oncology, Medical University of Vienna, Vienna, Austria; Chongqing University, CHINA

## Abstract

Cone beam computed tomography (CBCT) has become a vital tool in interventional radiology. Usually, a circular source-detector trajectory is used to acquire a three-dimensional (3D) image. Kinematic constraints due to the patient size or additional medical equipment often cause collisions with the imager while performing a full circular rotation. In a previous study, we developed a framework to design collision-free, patient-specific trajectories for the cases in which circular CBCT is not feasible. Our proposed trajectories included enough information to appropriately reconstruct a particular volume of interest (VOI), but the constraints had to be defined before the intervention. As most collisions are unpredictable, performing an on-the-fly trajectory optimization is desirable. In this study, we propose a search strategy that explores a set of trajectories that cover the whole collision-free area and subsequently performs a search locally in the areas with the highest image quality. Selecting the best trajectories is performed using simulations on a prior diagnostic CT volume which serves as a digital phantom for simulations. In our simulations, the Feature SIMilarity Index (FSIM) is used as the objective function to evaluate the imaging quality provided by different trajectories. We investigated the performance of our methods using three different anatomical targets inside the Alderson-Rando phantom. We used FSIM and Universal Quality Image (UQI) to evaluate the final reconstruction results. Our experiments showed that our proposed trajectories could achieve a comparable image quality in the VOI compared to the standard C-arm circular CBCT. We achieved a relative deviation less than 10% for both FSIM and UQI metrics between the reconstructed images from the optimized trajectories and the standard C-arm CBCT for all three targets. The whole trajectory optimization took approximately three to four minutes.

## 1. Introduction

Cone beam computed tomography (CBCT) has become a mainstay in interventional imaging with applications in image-guided surgery, interventional radiology, and image-guided radiotherapy [1–5]. Considerable research has focused on limited-angle or non-isocentric trajectories for CBCTs, motivated by either a reduction of dose [6–9] or spatial constraints [10–12]. The authors in [10] proposed a rotate-plus-shift C-arm trajectory that enables the acquisition of complete CT data with less than a 180° rotation. Their suggested method relaxes the requirement for a 180-plus fan-angle rotation and leads to a much broader use of intraoperative 3D imaging by reducing constraints on the C-arm geometry. Another study [11] proposed an accelerated implementation of an iterative method for CBCT reconstruction suitable for a limited angular span, which reduced computational time using GPU-accelerated kernels. That proposed scheme enables the use of advanced reconstruction methods that are needed in limited-data scenarios such as surgery. The authors in [12] introduced a CBCT verification method using unconventional and limited imaging angles for cancer patients undergoing non-coplanar radiation therapy. They illustrated that non-coplanar beams with coach rotations of 45° can be sufficiently verified with their CBCT acquisition technique.

Some studies have investigated patient-specific trajectories for CBCT acquisitions. Gang et al. [13] introduced a task-based imaging acquisition protocol for robotic C-arm CBCT systems using a gradient-based optimization of the reconstruction kernel, tube current, and orbital tilt (range: ±30°). Noncircular source-detector trajectories have been investigated using periodic and B-spline-based functions for simulation studies, as well as in neuroradiology applications [14, 15]. An increased image quality in a volume of interest (VOI) over the standard circular orbit was the main aim of these studies [13–15]. In a recent study, optimal sinusoidal trajectories were introduced to avoid the metal parts of the imaged object while still guaranteeing a high coverage in Radon space and its vicinity [16]. Another group [17] also introduced metal artifact reduction trajectories by computing a quality map of plausible views from the expected spectral shift, which is caused by beam hardening and depends on various path lengths of the photons passing through metal objects. All the aforementioned studies [13–17] were successfully applied to C-arm CBCT trajectory optimization. However, in all these approaches, hard constraints on the rotation angle were used for trajectory design; thus, the proposed trajectories did not incorporate patient-specific collisions. In addition, all these studies [13–17] computed optimal trajectory parameters in a (semi) offline manner.

In study [18], scene-specific collision-avoiding trajectories were introduced for linac-mounted CBCT systems using a virtual isocenter and variable magnification during acquisition. The proposed trajectories in that study achieved contrast and resolution comparable to that of a standard circular scan and would be suitable for patients who cannot be imaged with CBCT for image-guided radiation therapy because of their position, size, or fixation devices that could cause collisions with the gantry and the detector. Although their proposed approach could integrate case-specific collisions into the trajectory design, it requires a high amount of computational time which hampers its use for real-time trajectory optimization. Therefore, it is not suitable to react to unforeseen collisions that happen during interventions. To our knowledge, the only study that proposed real-time trajectory optimization was [19], in which the authors suggested optimizing the C-arm CBCT source-detector trajectory during the CBCT scan to improve reconstruction image quality in the presence of metal artifacts. They performed the adjustments on-the-fly using a convolutional neural network and regressed an image quality metric over all possible next projections given the current X-ray image. However, as the main focus of this study was metal artifact reduction, the proposed trajectories did not incorporate patient-specific collisions in their design. The research we present in this study is the first demonstration that introduces an on-the-fly trajectory optimization protocol for CBCT acquisition that is able to react to unpredictable collisions.

**Fig 1:**
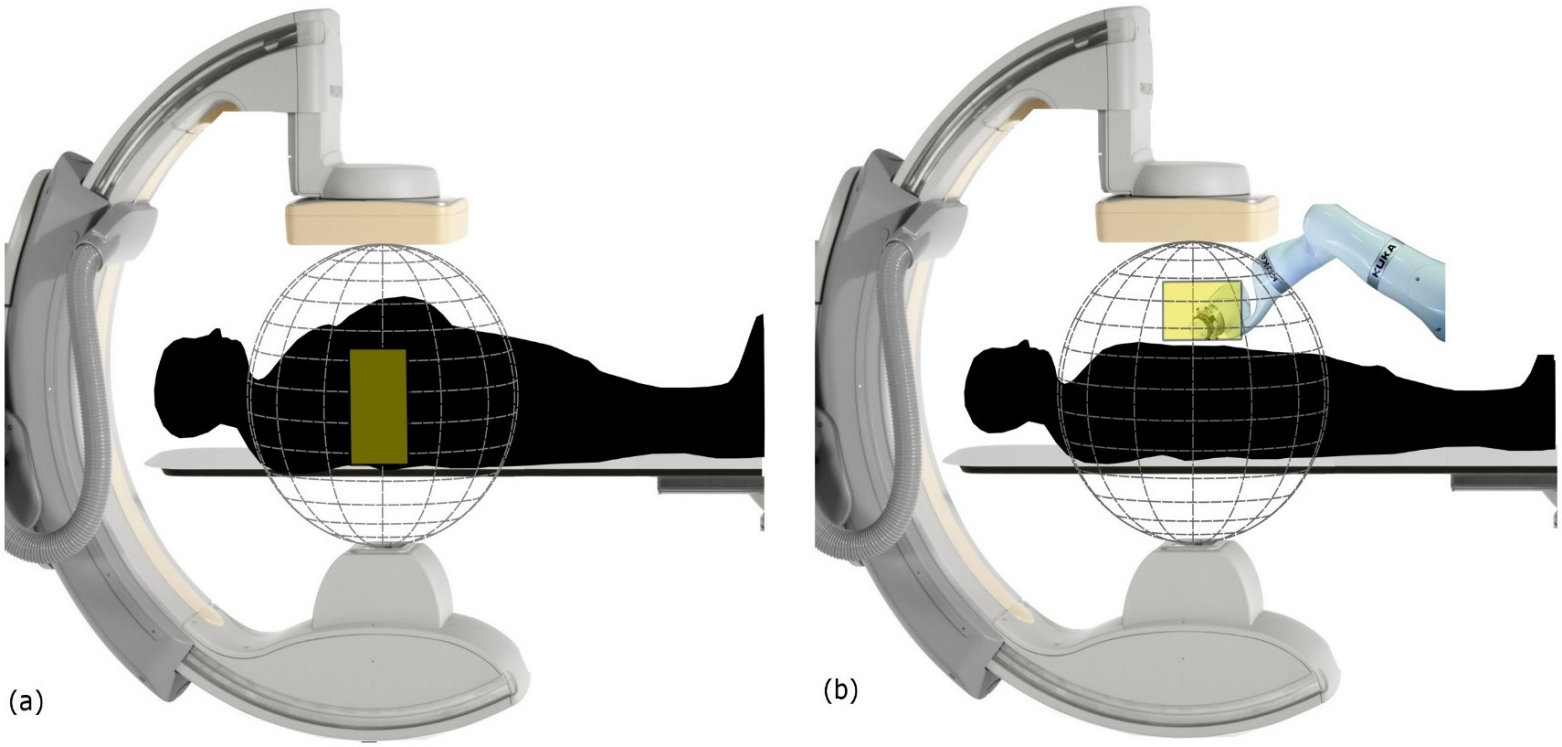
Two examples of kinematic constraints during interventions. Collision due to the patient size (a), and other medical devices (b).

The work basically builds on a recently published method to optimize imaging quality for CBCT using non-isocentric semi-circular scan trajectories which can also be arranged out-of-plane [8, 20, 21]. A volume-of-interest (VOI) is selected from a pre-existing diagnostic computed tomography (CT) scan, and a variety of possible trajectory combinations from short arcs is simulated while taking into account kinematic constraints. The optimal arc combination is selected based on the image quality within the VOI. The main purpose of the trajectory optimization in this study is to facilitate CBCT imaging in situations with strong kinematic constraints where standard circular trajectories are not feasible. While it is clear that such an approach cannot provide images of equivalent or better quality in the full field of view compared to a standard CBCT scan, which fully samples voxels near the central transverse plane, the main goal was to provide reasonable image quality in the selected VOI. We also note that our proposed optimization protocol is targeted to applications where repeated scans are required and a pre-operative high-quality scan exists. It is evident that this process is time-consuming. While it is possible to run the simulation and the associated trajectory selection process offline, the current time required to design a patient-specific imaging protocol was in the range of 80 minutes [20]. This required kinematic constraints to be known previously. As collisions are mostly unpredictable in a clinical scenario, e.g., caused by patient size or additional medical devices (Fig 1), a real-time trajectory optimization framework is of potential clinical importance even at the cost of a loss of image quality.

In this study, we propose a new search strategy to overcome the aforementioned computational limitation, which has the potential to optimize trajectories on-the-fly, and therefore, can take into account unforeseen collisions during interventions. We introduce a heuristic optimization procedure by identifying arcs with the greatest information to reconstruct the VOI, and consequently, perform a local search around those best initial candidate paths and investigate better arc solutions among newly created neighbors. The proposed search scheme requires identification of the collision-free trajectory area from a sensor input and searches among a reduced subset of possible arcs as the initial search space. Compared to our previous work [20], the main scientific novelty of this study lies in the introduction of a new search strategy that enables the on-the-fly feature for the path optimization scheme; this finally brings a significantly important clinical benefit for interventions where a 3D CBCT is otherwise not possible due to unpredictable collisions.

## 2. Materials and methods

### 2.A. Adaptation of workflow for the on-the-fly customized CBCT

We previously introduced a workflow to design patient-specific, source-detector trajectories for a CBCT imaging system [20]. In this study, we modified our methodology to integrate kinematic constraints caused during the interventions into the trajectory optimization process, which enables a dynamic optimization in the operating theater.

### 2.B. On-the-fly trajectory optimization method

**Fig 2:**
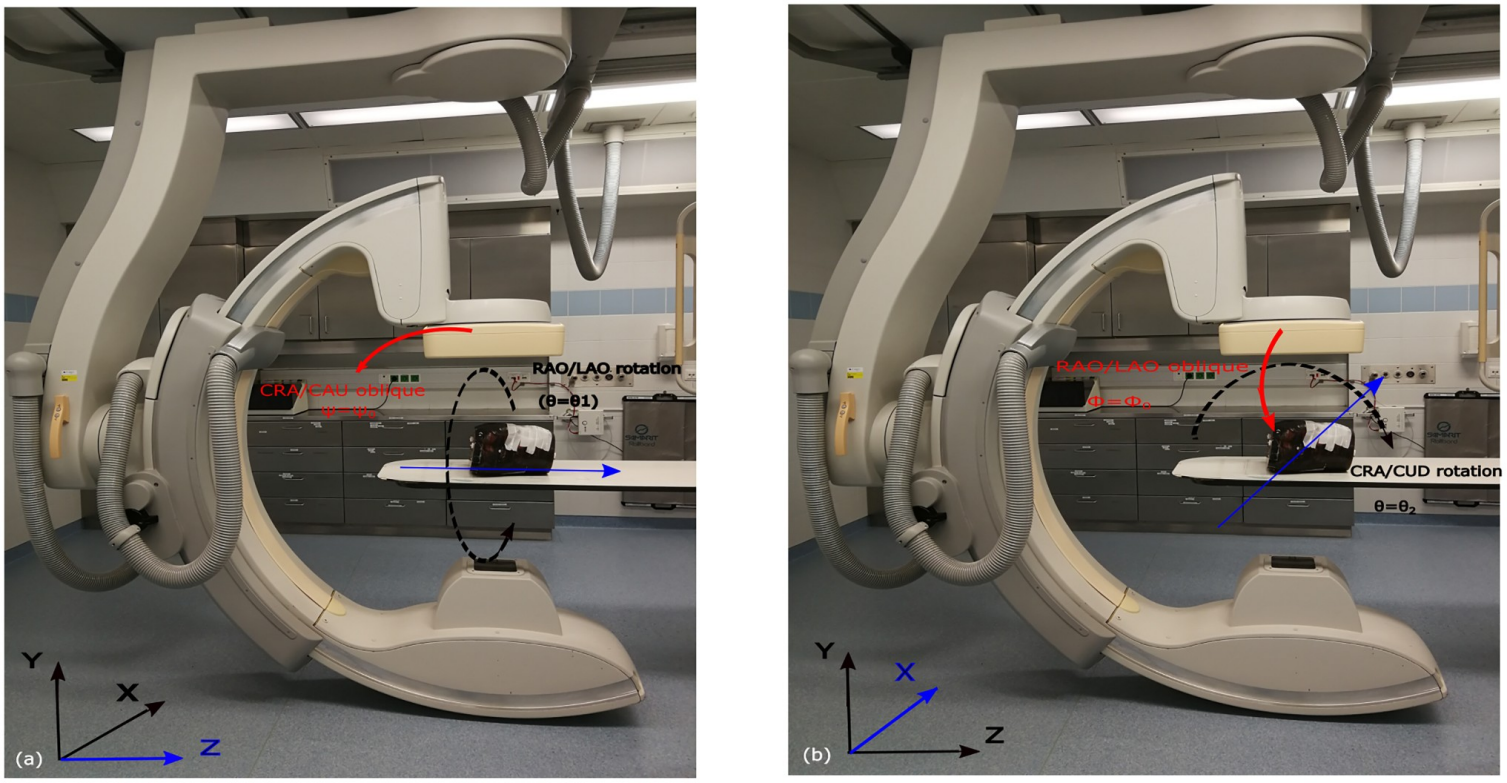
Philips Allura FD20 Xper C-arm possible rotations. (a) RAO/LAO rotation with cranial CRA/ CAU tilt, (b) CRA/CAU rotation with RAO/LAO tilt. Rotation axes for RAO/LAO and CRA/CAU rotations are Z and X, respectively, which are shown in the blue color.

**Fig 3:**
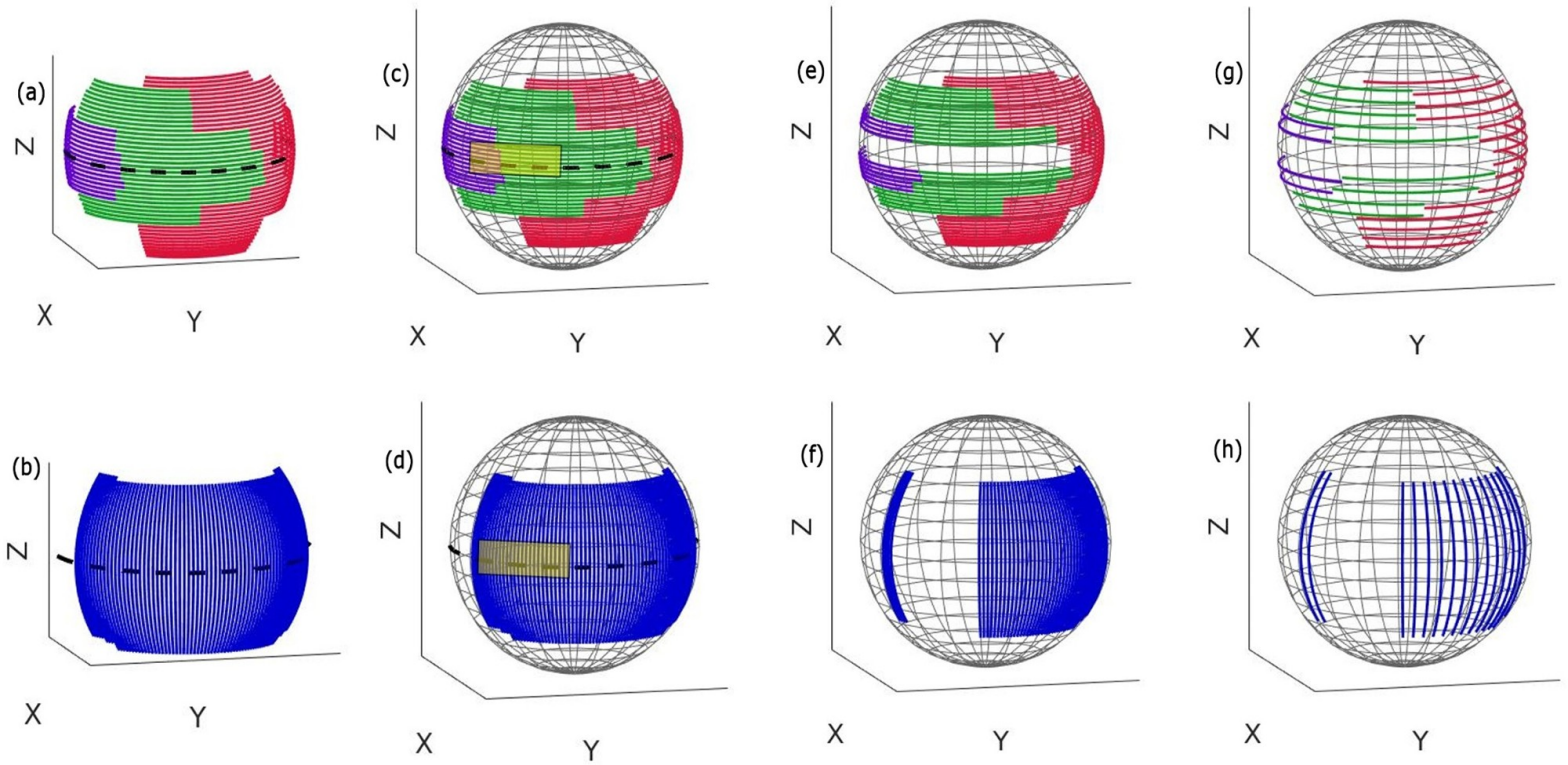
Possible rotations are divided into arcs with less than 80°. (a) RAO/LAO arcs with CRA/CAU obliques shown in the purple, green, and red colors, (b) CRA/CAU arcs with RAO/LAO obliques shown in the blue color, (c) and (d) spherical plot of arcs with a forbidden area, (e) and (f) spherical plot of the arcs after removing those that intersected the forbidden area, (g) and (h) spherical plot of these remaining arcs after sparsification. Only these arcs were in the search space for trajectory optimization. (Kinematic constraints are simulated as forbidden areas on the geometry of the system and are shown as yellow rectangles.).

**Fig 4:**
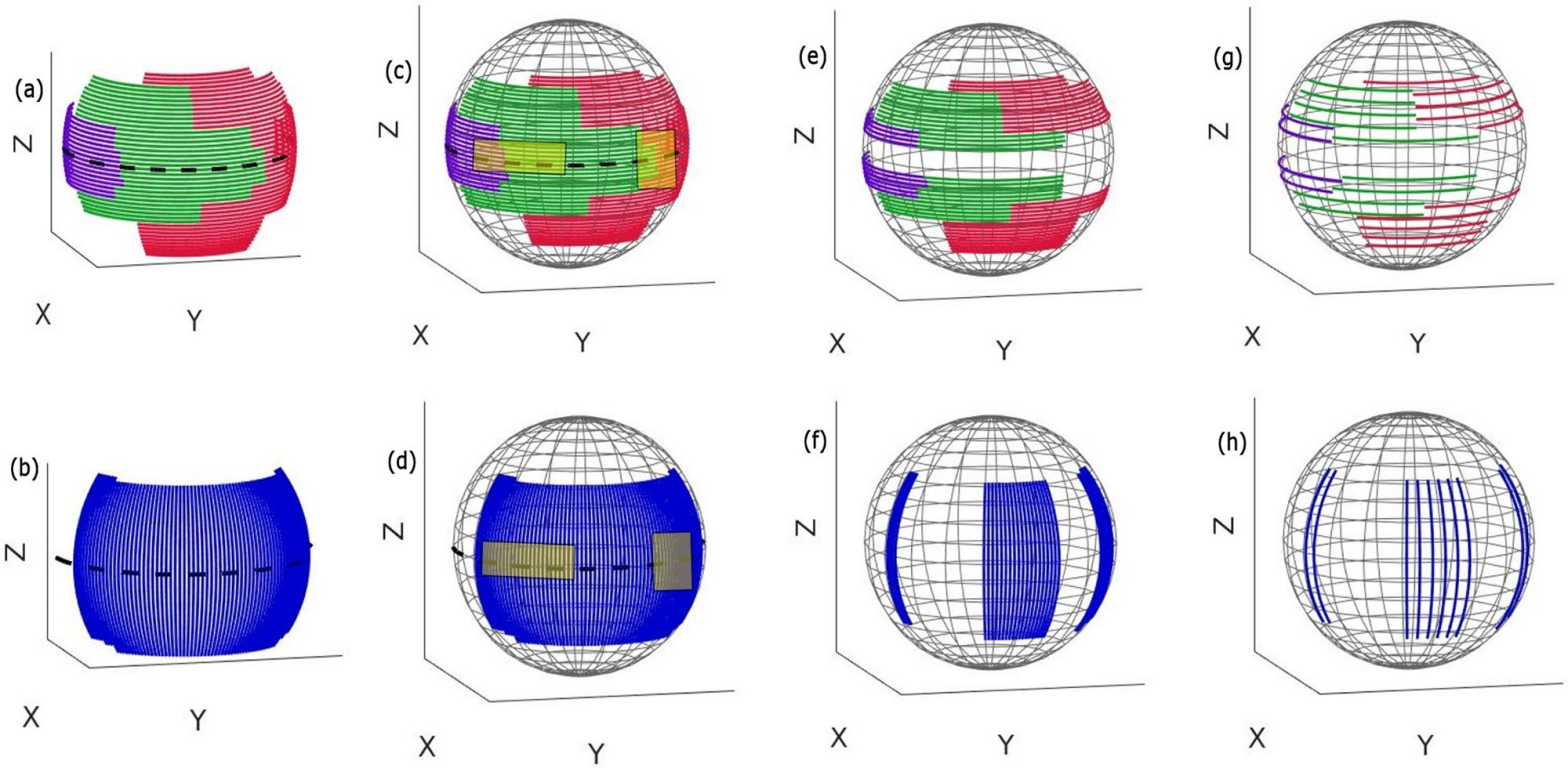
Possible rotations are divided into arcs with an 80° maximum and are shown in different colors. (a) RAO/LAO arcs with CRA/CAU obliques shown in the purple, green, and red colors, (b) CRA/CAU arcs with RAO/LAO obliques shown in the blue color, (c) and (d) spherical plot of arcs with an illustration of two forbidden areas as the area inside the yellow rectangles, (e) and (f) spherical plot of the residual arcs after removing those which were inside the forbidden area, (g) and (h) spherical plot of the residual arcs after sparsification. Only those remaining arcs were included in the search space for our trajectory optimization. (Kinematic constraints are simulated as forbidden areas on the geometry of the system and are shown as yellow rectangles.).

In this study, the geometry of the Philips Allura FD20 Xper C-arm is used to define the set of possible arcs. This C-arm can perform two types of rotations: 1) It is able to rotate by angle θ_1_ towards the Right Anterior Oblique (RAO)/Left Anterior Oblique (LAO) direction while having a tilt ψ at different fixed Cranial (CRA)/Caudal (CAU) angles (Fig 2a). It is also able to rotate by angle θ_2_ towards the CRA /CAU direction while having a tilt φ at different fixed RAO/LAO angles (Fig 2b). Arc definition is similar to that in the previous work [20]: two subset of arcs were defined (Figs 3, 4a and 4b), each corresponding to a rotation on the Philips Allura, rotation on RAO/LAO (Figs 3 and 4a) and CRA/CAU directions (Figs 3 and 4b). In each of those directions, the C-arm can have oblique angles, Ψ and Φ, which define the rotation on that direction of the arc. Each arc included less than 80 projections, which were sampled every degree. During interventions, kinematic constraints due to other medical devices and patient size may occur (as shown in Fig 1a and 1b). In this study, we simulated such kinematic constraints as forbidden areas on the geometry of the C-arm system (shown as yellow rectangles in Figs 3, 4c and 4d).

We removed arcs that had more than 10% of their angular range in the forbidden area and cropped those that had less than 10% in it, Figs 3, 4e and 4f. To accelerate further the optimization process, we modified our previous approach by sparsifying the initial subset of arcs (Figs 3, 4e and 4f) to include just arcs for every six degrees (Figs 3, 4g and 4h). This step will significantly reduce the brute-force search space, and therefore, lead to a significant reduction in the computation time. However, a reduction of the initial subset of arcs may introduce an unfavorable bias in the trajectory selection process. To address this issue, we performed a heuristic local search around the arcs with the highest amount of information. In fact, the major difference from the previous approach [20] is that we concentrated on the most informative areas in 3D space to reconstruct the VOI (rather than searching among all plausible arcs as proposed in our previous study [20]), and consequently, we performed a local search around those selected optimal areas to find a better arc solution. In detail, first, the three arcs with the best objective function values were selected as the arcs with the greatest amount of information. Afterward, we created new neighbor arcs for each of these three selected arcs and searched through such nearest neighbor arcs until we observed an improvement in the objective function. Finally, the arc with the highest objective function was selected. This procedure was repeated for the arc subset RAO/LAO and CRA/CAU one after the other until a predefined number of arcs was selected as the final trajectory.

**Fig 5:**
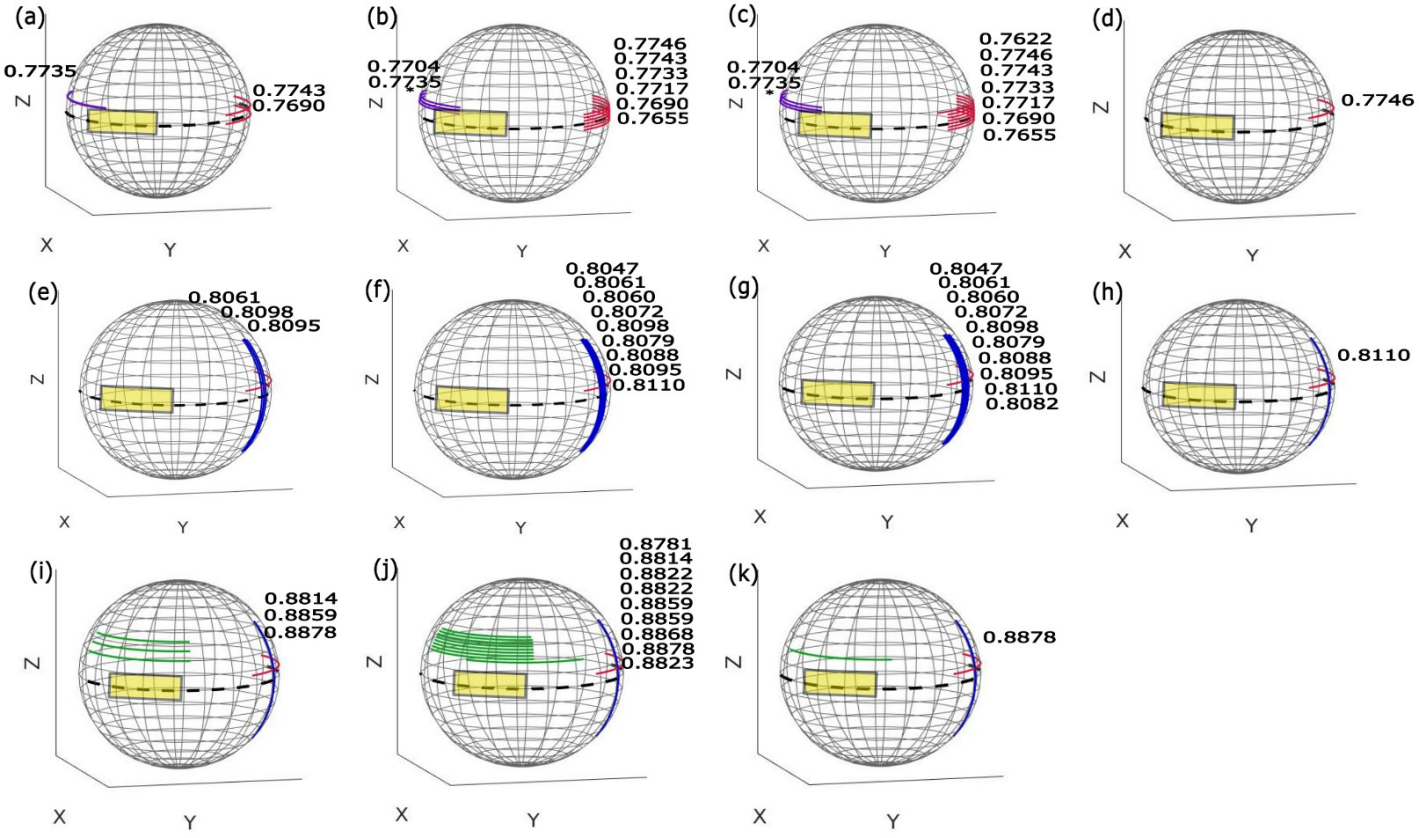
Illustration of the search strategy proposed for the on-the-fly trajectory optimization, with the optimized trajectory that included three arcs selected for the neck target with one forbidden area simulation. (a-d) Optimizing the first best arc, (e-h) optimizing the second best arcs, and (i-k) optimizing the third best arc. The number close to each arc shows the value of FSIM achieved related to that arc. The sign (*) shows that the arc included more than 10% of its angular range in the forbidden area, and therefore, was rejected from the search space and FSIM was not calculated.

We illustrate the procedure of arc selection with an example of the neck image target while simulating one forbidden area in Fig 5. For arc selection, we first searched through the RAO/LAO arc sparsely sampled initial subset (Fig 3g) and we selected the three neighboring arcs (every one degree) with the highest objective function value (Fig 5a). Then, we searched the nearby arcs until the objective function decreased (Fig 5b and 5c). The best arc from this optimization was then selected for the final trajectory (Fig 5d), and the optimization changes to the CRA/CAU subset of arcs, while the previous best arc was still being used, were prepended onto the CRA/CAU arcs (Fig 5e). This process was then repeated for the second arc subset (Fig 3h) in such a way that the combination of two arcs would give us the best objective function value (Fig 5e–5h). This process can be repeated for arc subsets RAO/LAO and CRA/CAU successively as many times as the user requires. In this example, we optimized for a third arc on the RAO/LAO subset as well (Fig 5i–5k). As the objective function, we used the value of Feature SIMilarity Index (FSIM), as in our previous study [20]. The pseudocode for this procedure is presented in Algorithm 1.

Algorithm 1. Trajectory optimization

**Input**: Search space, number of desired arcs

**Step 1**: Simulate projections for all defined arcs with the digital phantom

**Step 2: FOR** 1: number of subsets

   **Step 3: FOR** 1: number of arcs in subset

-   Reconstruct the image using the set of projections related to the corresponding arc
-   Crop the reconstructed image at the VOI
-   Calculate the objective function at the cropped area

  **END**

 **Step 4**: Select best three arcs from Step3

 **Step 5: WHILE** expanded arcs increase objective function

   **Step 6: FOR** 1:number of arcs to expand

-     Create neighboring arcs at one degree each side
-     Evaluate objective function in newly created neighbors

   **END**

 **END**

 **Step 7**: Select best arc and prepend to search space

 **END**

**Step 8**: Return selected trajectory (combined arcs)

### 2.C. Image reconstruction

We used a modified version of the TIGRE toolkit for arbitrary trajectories similar to [20–22], but with the Adaptive Steepest Descent Projection Onto Convex Sets (ASD-POCS) reconstruction limited to five iterations.

Projections were sampled every four degrees, and therefore, 20, 40, and 60 projections were simulated for trajectories that included one, two, and three arcs, respectively. We note that this projection number reduction was done only in simulations for a further acceleration of the optimization method; however, for the real data, the full sampling projections were used for the reconstruction.

### 2.D. Optimization of computational time

We modified the implementation of ASD-POCS in the TIGRE toolbox to run the reconstruction on the GPU. This implementation takes approximately 1.4, 2.2, and 3.05 seconds for each ASD-POCS reconstruction (with five iterations), including 20, 40, and 60 projection angles using a computer with an NVIDIA GeForce RTX 2080 and a 32-core Advanced Micro Devices (AMD) processor. The total number of RAO/LAO arcs after sparsification (Figs 3 and 4g) was 28 and 23, respectively, using one and two simulated forbidden areas. In addition, the total number of CRA/CAU arcs after sparsification (Figs 3 and 4h) was 15 and 10, respectively, using one and two simulated forbidden areas. We used 256^3^ voxel volumes with 512^2^ projections for the reconstruction. Optimizing the first optimal arc (including 20 projections) required 28 and 23 reconstruction runs, which took approximately 39 and 32 seconds, respectively, while one and two simulated forbidden areas were simulated. In addition, optimizing the second optimal arc (including 40 projections) required 15 and 10 reconstruction runs, which took approximately 33 and 22 seconds for one and two simulated forbidden areas, respectively. Finally, optimizing a third optimal arc (including 60 projections) required 28 and 23 reconstruction runs, which took approximately 84 and 70 seconds for one and two simulated forbidden areas, respectively. The overall time needed for the whole procedure, including also the calculation of the objective function and projection simulations, was approximately three to four minutes. The numbers reported in this study used one GPU for reconstruction.

### 2.E. Evaluation

#### 2.E.1. Phantoms

**Fig 6:**
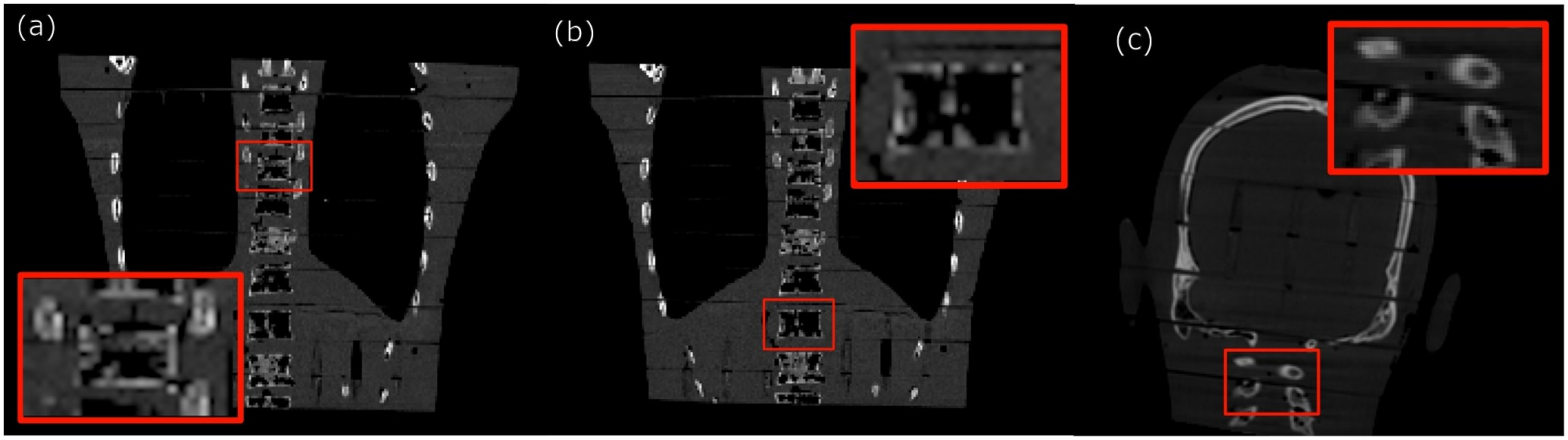
Three VOI selected for our experiments, (a) T3/T4 regions of the thoracic spine (Target 1), (b) T10/T11 regions of the thoracic spine (Target 2), and (c) C1/C2 region of the cervical spine (Target 3).

We evaluated three imaging targets in two regions of the Alderson-Rando phantom. Target 1 and Target 2 were considered the two VOIs in the T3/T4 (Fig 6a) and T10/T11 (Fig 6b) regions of the thoracic spine, respectively. Target 3 was considered the VOI in the C1/C2 region of the cervical spine (Fig 6c).

#### 2.E.2. Experiments

CT scan parameters for the Alderson phantom were 120 kV, 350 mAs, and 1 mm^3^ voxel size, on a SOMATOM Definition AS, Siemens Healthineers, Erlangen, Germany. For each target, we optimized trajectories for two and three arcs, with one or two forbidden areas each. Using a step-and-shoot protocol, the optimal trajectories were implemented. The reconstruction results were then compared to the C-arm standard circular trajectory (313 projections, 210° angular range). In addition, they were also compared to a reconstruction from a simple, partial circular trajectory with an angular range and projections equivalent to the optimized trajectory. For all reconstructions, we used the ASD-POCS and evaluated using FSIM and Universal Quality Image (UQI). For both metrics, the image quality index between the C-arm standard circular CBCT and the prior CT was considered the reference value. The quality index value between the optimized/partial circular trajectory and the prior CT was considered as the measured value. The relative deviation between the reference and measured values was used for the image quality evaluation.

## 3. Experimental results

We display the reconstructed images and the optimized trajectories only for Target 1 (Fig 6a), but all the quantified results can be found in the tables.

### 3.A. Optimized trajectories

**Fig 7:**
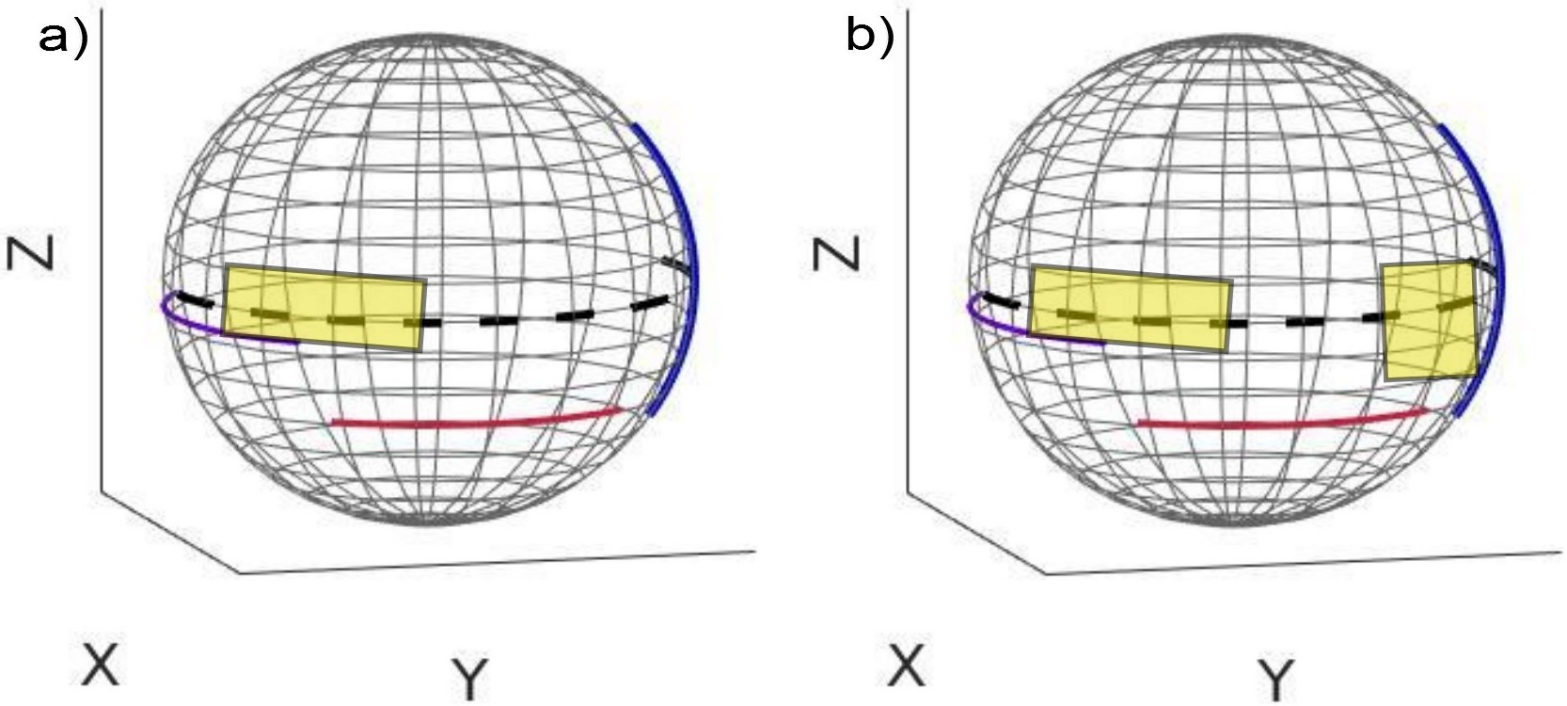
3D visualization of the optimized trajectories with respect to the C-arm circular trajectory for the Target 1 with one simulated forbidden area (a) and two simulated forbidden areas (b). (Trajectories were the same when simulating one or two forbidden areas).

**Fig 8:**
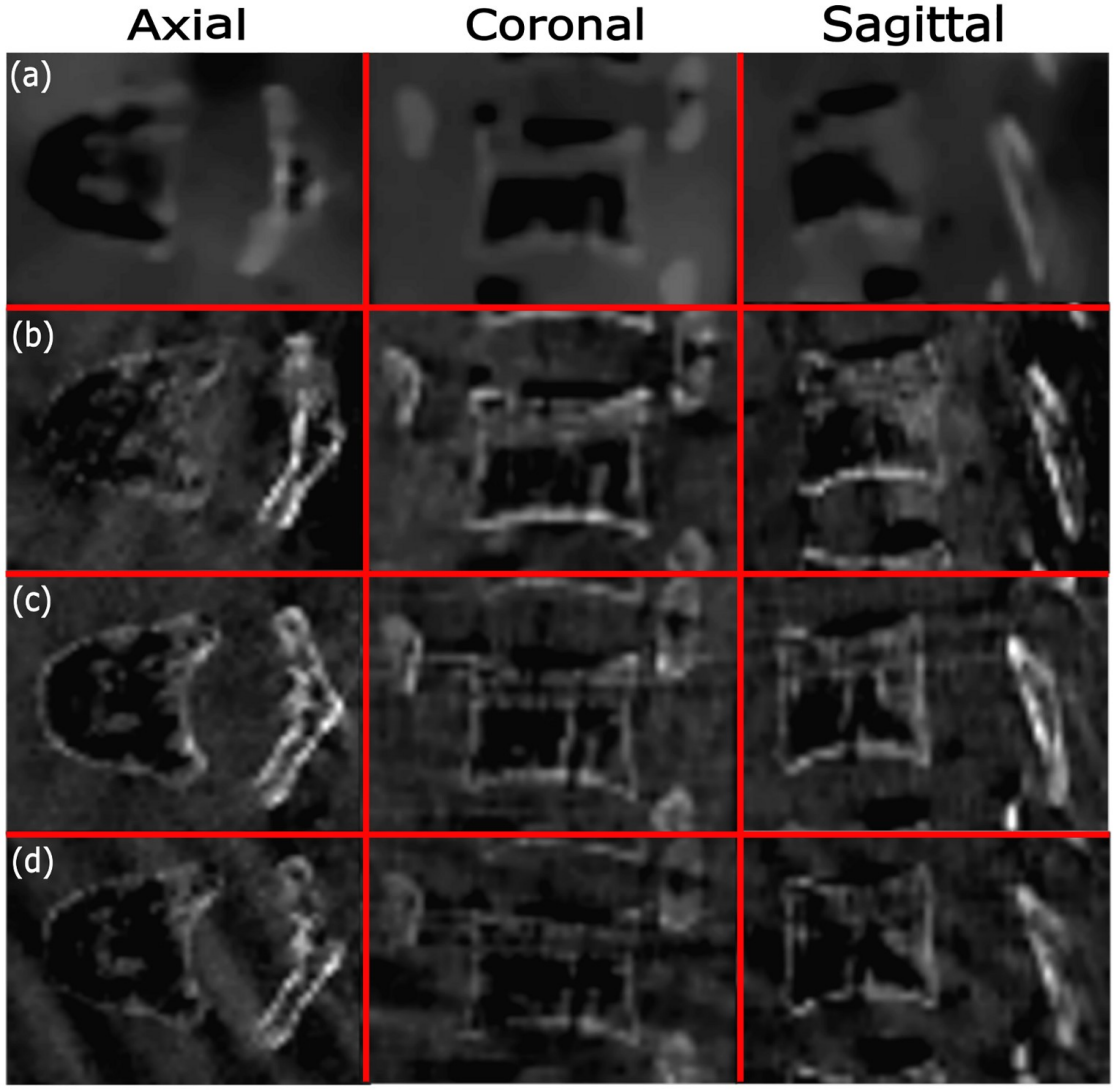
Reconstructions with one forbidden area (the reconstructions are also the same for two forbidden areas, as the trajectories happened to be the same) related to Target 1 using (a) optimized trajectory that included two arcs based on simulation data, (b) optimized trajectory that included two arcs based on real data, (c) C-arm circular trajectory based on real data, and (d) partial circular trajectory based on real data. The display window uses the range 200–3000 HU for (a), and a range of 0–21 in gray values for (b-d), respectively.

**Fig 9:**
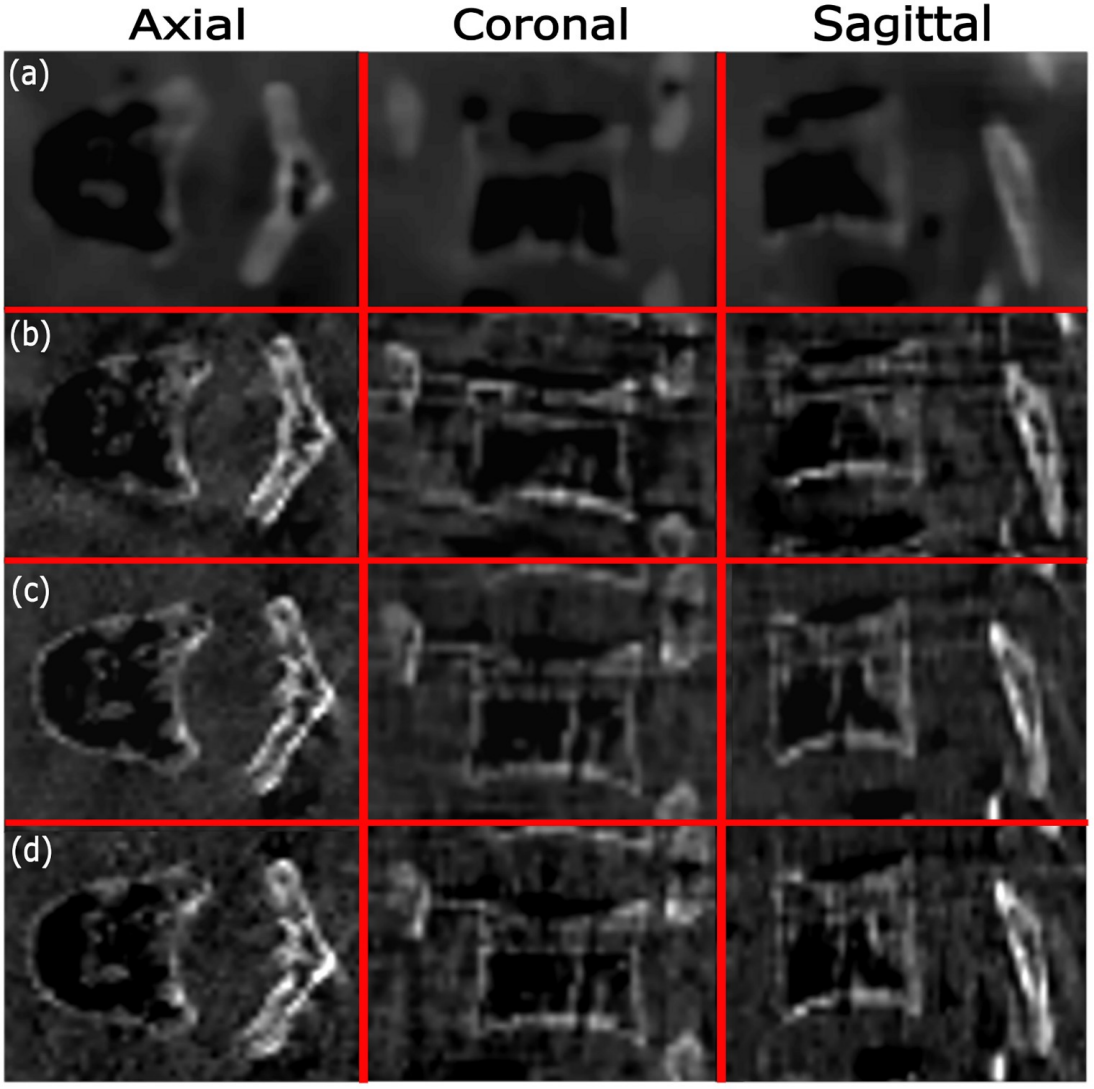
Reconstructions with one forbidden area (the reconstructions are also the same for two forbidden areas, as trajectories happened to be the same) related to Target 1 using (a) optimized trajectory that included three arcs based on simulation data, (b) optimized trajectory that included three arcs based on real data, (c) C-arm circular trajectory based on real data, and (d) partial circular trajectory based on real data. The display window uses a range of 200–3000 HU for (a) and a range of 0–21 in gray values for (b-d), respectively.

3D visualizations of the optimized trajectories for one and two simulated forbidden areas for Target 1 are shown in Fig 7a and 7b, respectively. Angular range and projection numbers related to optimized trajectories selected for all targets can be found in Tables 1 and 2. The (-) sign denotes rotation to the right/caudal directions and the (+) sign denotes rotation to the left/cranial, both with respect to the patient reference position. For Targets 1 and 2, the final trajectories were the same when using one or two simulated forbidden areas, while for Target 3 different trajectories were achieved when simulating one or two forbidden areas (Tables 1 and 2). The reconstructed images for Target 1 based on simulation data using optimized trajectories that included two and three arcs, are found in Figs 8a and 9a, respectively.

**Table 1:** The angular range and projection number of the three selected arcs for the optimized trajectories related to both thorax targets using one simulated forbidden area, and total of projections number for the final optimized trajectories related to both Target 1 and Target 2. (For both Target 1 and Target 2, the three selected arcs were the same when considering one or two simulated forbidden areas; therefore, the numbers reported here are the same when using two forbidden areas.).

Trajectory	Arc	Angle	Projection number per arc	Total number of projections
**Target 1**	Arc 1	θ_1_ = -39:1:+39, ψ = -26	72	228
	Arc 2	θ_2_ = -34:1:+40, φ = -60	75	
	Arc 3	θ_1_ = +44:1:+124, ψ = -6	81	
**Target 2**	Arc 1	θ_1_ = -22:1:+50, ψ = 10	73	227
	Arc 2	θ_2_ = -40:1:+38, φ = -50	79	
	Arc 3	θ_1_ = +9:1:+83, ψ = +32	75	

**Table 2:** The angular range and projection number of the three selected arcs for the optimized trajectories related to Target 3 using one or two simulated forbidden areas. (For Target 3, the three selected arcs were different when considering one or two simulated forbidden areas.).

Trajectory	Arc	Angle	Projection number per arc	Total number of projections
**Target 3**	Arc 1	θ_1_ = -110:1:-34, ψ = + 6	77	234
**One forbidden area**	Arc 2	θ_2_ = -39:1:+38, φ = -22	78	
	Arc 3	θ_1_ = +8:1:+86, ψ = 18	79	
**Target 3**	Arc 1	θ_1_ = +49:1:+124, ψ = 10	76	234
**Two forbidden areas**	Arc 2	θ_2_ = -40:1:+38, φ = -18	79	
	Arc 3	θ_1_ = -39:1:+39, ψ = +42	79	

### 3.B. Reconstructed images

For both Target 1 and Target 2, the three selected arcs were the same when considering one or two simulated forbidden areas; therefore, the reconstructed images and also the computed image quality metrics were the same when using one or two forbidden areas (Table 3). For Target 3, however, two different trajectories were selected when simulating one or two forbidden areas; therefore, the calculated image quality metrics were different for one or two forbidden area simulations (Table 4).

**Table 3:** Relative deviations (%) of image quality measures FSIM and UQI related to Target 1 and Target 2 for both optimized and partial circular trajectories using one forbidden area (for both Target 1 and Target 2, the three selected arcs were the same when considering one or two simulated forbidden areas; therefore, the reconstructed images and the computed image quality metrics reported here are the same when using two forbidden areas).

		Image quality metric	Trajectory	Two arcs	Three arcs
**Relative deviation (%)**	**Target 1**	**FSIM**	**Opt**.	13.90	9.47
			**Partial-circ**.	15	7.87
		**UQI**	**Opt**.	13.57	8.49
			**Partial-circ**.	16.77	4.83
	**Target 2**	**FSIM**	**Opt**.	8.04	3.90
			**Partial-circ**.	8.30	5.39
		**UQI**	**Opt**.	10.47	4.06
			**Partial-circ**.	12.45	5.38

**Table 4:** Relative deviation (%) of image quality measures FSIM and UQI related to Target 3 for both optimized and partial circular trajectories using one and two simulated forbidden areas.

		Image quality metric	Trajectory	Two arcs	Three arcs
**Relative deviation (%)**	**Target 3**	**FSIM**	**Opt**.	11.01	5.90
			**Partial-circ**.	12.94	3.98
	**One forbidden area**	**UQI**	**Opt**.	16.08	8.67
			**Partial-circ**.	17.42	7.48
	**Target 3**	**FSIM**	**Opt**.	10.86	5.69
			**Partial-circ**.	12.94	3.98
	**Two forbidden areas**	**UQI**	**Opt**.	16.26	9.93
			**Partial-circ**.	17.42	7.48

#### 3.B.1. Reconstructed images using two arcs

Reconstruction results based on real data for the optimized trajectories that included two arcs, standard C- arm circular, and partial circular trajectories related to Target 1, are shown in Fig 8b–8d. Relative deviation of the image quality measures FSIM and UQI related to the optimized trajectories that included two arcs and also partial circular trajectories for all three targets, are given in Tables 3 and 4. According to the results of Table 3, for both FSIM and UQI metrics, the reconstructed image related to the optimized trajectory that included two arcs, showed a relative deviation up to 13.90% and 10.47% for Target 1 and Target 2, respectively. We also achieved a relative deviation up to 16.77% and 12.45% for Target 1 and Target 2, respectively, for the reconstructed images related to partial circular trajectories. In addition, according to the results of Table 4, for both FSIM and UQI metrics, the reconstructed image related to the optimized trajectory that included two arcs for Target 3, using one and two simulated forbidden areas, represents the relative deviation up to 16.08% and 16.26%, respectively. For the reconstructed images related to partial circular trajectories, a relative deviation up to 17.42% was achieved for Target 3 with one and two forbidden areas. According to the results of Tables 3 and 4, a lower relative deviation using both FSIM and UQI metrics was achieved for all three targets when using optimized trajectories that included two arcs, compared to the partial circular trajectory. These results show that the reconstruction results related to optimized trajectories that included two arcs for all three targets, slightly increased the image quality in the VOI compared to the partial circular trajectory.

#### 3.B.2. Reconstructed images using three arcs

Reconstruction results based on real data for the optimized trajectories that included three arcs, standard CBCT, and partial circular trajectories are shown in Fig 9b–9d. Relative deviation of the image quality measures FSIM and UQI related to the three targets for optimized trajectories that included three arcs and partial circular trajectories are also given in Tables 3 and 4. The optimized trajectories delivered relative deviations up to 9.47% and 4.06% in both image quality metrics for Target 1 and Target 2, respectively (see Table 3). A relative deviation up to 7.87% and 5.39% for Target 1 and Target 2, respectively, was also calculated for the reconstructed images related to partial circular trajectories. In addition, the results of Table 4 show that, for both FSIM and UQI metrics, the reconstructed image related to the optimized trajectories that included three arcs for Target 3, including one and/or two simulated forbidden areas, represents the relative deviation up to 8.67% and 9.93%, respectively. For the reconstructed images related to partial circular trajectories, a relative deviation up to 7.48% was achieved for Target 3 with one and/or two forbidden areas. According to the results of Tables 3 and 4, we observed a slightly higher relative deviation using both FSIM and UQI metrics for Target 1 and Target 3 (for both one and two forbidden areas) when using optimized trajectories that included three arcs compared to the partial circular trajectories. In contrast, a slightly lower relative deviation using both FSIM and UQI metrics for Target 2 was achieved when using optimized trajectories that included three arcs compared to the partial circular trajectory. These results show a small decreased reconstruction performance for Target 1 and Target 3, while a small increased image quality for Target 2 can be seen when using optimized trajectories that included three arcs compared to the partial circular trajectory. Furthermore, we observed almost similar relative deviation for both image quality metrics for Target 3 using optimized trajectories (including both two and three arcs) when simulating two forbidden areas compared to one forbidden area. In addition, when comparing the reconstruction results achieved for optimized trajectories that included two and three arcs, we observed a decrease in the relative deviation related to three-arc optimized trajectories compared to two-arc optimized trajectories for all three targets.

## 4. Discussion and conclusion

We introduced a framework for a patient-specific trajectory design for CBCT imaging under strong kinematic constraints. The proposed framework has the potential to be done on-the-fly; therefore, this framework is considered suitable for interventions with arbitrary and unexpected collisions. The authors in [10, 11] proposed generic trajectories and reconstruction implementations suitable for data with an angular range less than 180°. However, compared to our study, their trajectories were not patient-specific and could not incorporate case-dependent collisions in the trajectory design. The proposed approach in [18] could incorporate scene-specific collisions and the angular constraints due to gantry, patient, couch, and an onboard imaging system for linac-mounted CBCT systems in the patient trajectory design, but the drawback of their approach is that it requires angular constraints and collisions to be known in advance and cannot incorporate unexpected collisions in the path optimization on-the-fly due to the high computation demands. We note that, in [18], which is the most similar approach to our solution in terms of customized collision avoidance trajectory design, comparable reconstruction results with respect to standard circular scan were achieved. In this study, we also achieved comparable image quality compared to the reference circular CBCT; however, a comparison of the image quality of our method with that study [18] is not possible due to the completely different image quality metrics used. In addition, the only conceptually similar study to our approach in terms of on-the-fly trajectory optimization is [19], which proposes a near-online trajectory optimization design; however, the focus of their approach is to reduce metal artifacts, and unforeseen collisions cannot be incorporated in their trajectory design. To our knowledge, our study is the first demonstration of the feasibility for the design of scene-specific, noncircular CBCT trajectories that are suitable to react to unforeseen collisions.

Our results, based on both head and thorax targets, showed that optimized trajectories could achieve an image quality comparable to that of the reference circular CBCT for a given VOI. We investigated optimized trajectories that included two and three arcs. We observed a reasonable image quality for thorax targets, even using trajectories with two arcs. Considering that each arc includes around 80 projections and 80°, the optimized trajectory with two arcs includes approximately 160 projections and 160°. The two-arc trajectories employ limited-angle view data with at least 50° less and 150 projections compared to standard C-arm CBCT, which includes 313 projections and a 210° angular range. This makes our proposed trajectories suitable for a low-dose and limited-angle CBCT reconstruction. In addition, we observed the optimized trajectories with three arcs (with, at maximum, 234 projections and 234°) achieved an image quality comparable to that of the standard C-arm CBCT for all three targets. We achieved a relative deviation of less than 10% for both the FSIM and UQI metrics using optimized trajectories that included three arcs for all targets. In our previous study [14], relative deviations less than 7% were achieved for both FSIM and UQI for one target. In this study, we evaluated the results on three targets and observed a slight reduction in image quality. We note that, in our new search method the reduction of initial arc space helps to accelerate the optimization process, but this previously limited arc search space can cause neglect of good plausible arcs. Although the proposed heuristic local search performed around the newly created neighbor arcs compensates for this inefficiency to some extent and avoids skipping such good arcs, this limited trajectory space still affects the efficiency of the reconstructed results and caused a slight degradation of the image quality compared to our previous approach [20]. However, considering that the trajectory optimization can now be done on-the-fly, the reconstruction performance achieved in this study seems sufficient. In a clinical application, one could start with optimization using two arcs and, if there are higher image quality demands at the VOI, search for a third optimized arc.

We observed different relative deviations related to the reconstructed images of different targets (Tables 3 and 4). Image quality variation over different VOIs in the reconstructed image is common in circular CBCT due to different locations, voxel values, total attenuation path along the X-rays, etc. This effect is, nevertheless, not omitted when using optimized paths and is considered the reason for different relative deviation calculated for different targets in this study. For all three targets, the reconstruction results related to optimized trajectories that included two arcs showed an image quality improvement compared to the partial circular trajectory using both the FSIM and UQI index. The improvement could be due to the limited-angle artifact reduction (streaking artifacts appear less in Fig 8b compared to Fig 8d) achieved by the optimized trajectory compared to the partial circular trajectory. For optimized trajectories that included three arcs, the reconstructed image related to Target 2 showed a small image quality improvement compared to a partial circular trajectory, while the reconstruction results related to Target 1 and Target 3 showed a small image quality degradation compared to the partial circular trajectory. However, the differences are not significant and images reconstructed from optimized trajectories that included three arcs exhibited a comparable image quality for all three targets with regard to the partial circular trajectories. Considering the fact that our approach is the first proposed protocol in the literature that can facilitate CBCT for interventions in which a 3D circular CBCT would not be possible otherwise due to unpredictable collisions, our results are still significant even if there is a slightly image quality reduction for some targets compared to the partial circular trajectory.

In this study, we simulated both one and two forbidden areas to evaluate the performance of the optimization protocol under strong kinematic constraints. We observed a rather similar relative deviation for image quality metrics calculated for Target 3 using optimized trajectories when simulating one and two forbidden areas. This result shows that the optimization performance and the achieved image quality was not influenced significantly when simulating more forbidden areas, and therefore, embracing the robustness of our optimization protocol under kinematic constraints. In addition, when comparing the reconstruction results achieved for optimized trajectories that included two and three arcs, we observed a decrease in the relative deviation (up to 7.41%) when using three-arc optimized trajectories compared to two-arc optimized trajectories. However, such a result was expected, as a larger angular span and more projection numbers were used in the optimized paths that included three arcs compared to trajectories with two arcs.

The heuristic local search proposed in this study helps to find global maxima (because it is not fixed into a choice once selected) while selecting among only limited initial arc solutions rather than searching among all possible paths, as introduced in our previous study [20]; this reduces the total computational time of the arc-selection process significantly. The trajectory optimization framework requires three to four minutes overall time on one GPU and a further reduction in time to around one minute is expected by using multiple GPUs. Compared to our previous work [20], which required approximately 80 minutes for reconstruction, we have achieved a considerably higher speed in this study.

In our previous study [20], we proposed to combine short arcs (in both RAO/LAO and CRA/CAU directions). This allowed for additional degrees of freedom compared to a single-view arc and could enhance reconstruction performance compared to just a continuous limited view single arc. Limited angle trajectories with less than 180° are efficient for a wide variety of applications [10–12, 23, 24]. We selected a maximum 80° for each arc to avoid a final trajectory with two arcs that would exceed 160°. Our previous [20] and also the current study show reasonable reconstructed results for different VOIs while using two 80° arcs as the final trajectory. However, our main purpose in dividing the trajectory into short 80° arcs (two or three arcs instead of a full circular trajectory) was to allow for an increased flexibility under inevitable kinematic constraints. This facilitates CBCT under severe kinematic constraints, for example, when arcs larger than 80° are not possible due to collisions. We note that the length of each arc was selected with our constraints, but other options can also be selected. In fact, our proposed strategy can be fully used to design a multi-arc trajectory by selecting the number of arcs and the length per arc in advance, which is especially relevant in C-arm-based CBCT implementations and applications.

Our proposed trajectory optimization protocol can easily be applied to other imaging devices with general source-detector trajectories and additional degrees of freedoms. In this case, non-isocentric trajectories can also be optimized similar to the proposed framework. Extension of our methodology for field-of-view enlargement/shift using such non-isocentric trajectories is a future perspective of our work. Sparing the organ-at-risk (OAR) is standard in radiotherapy dose-planning, but has not yet been considered for pre-fractional position verification imaging. An important point of future work is to perform dose-planning for CBCT imaging. This is feasible based on our trajectory optimization methodology by determining the optimized orientation in 3D space and skipping projections, which deliver high doses to the OAR.
